# Supplementary material for: Robust minimally-invasive microfabricated stainless steel neural interfaces for high resolution recording
Source: Nat Commun. 2026 Jan 9;17:957. doi: 10.1038/s41467-025-67681-w (PMC12848124; doi:10.1038/s41467-025-67681-w)
Supplement: Supplementary file 1 — Supplementary Information [file 41467_2025_67681_MOESM1_ESM.pdf]

Supplementary Information

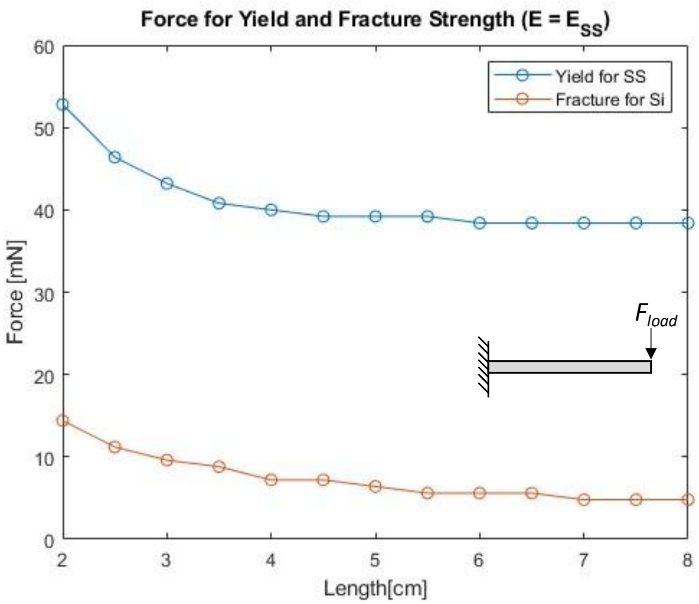

Figure S1. **Forces required for fracturing Silicon (Si) probe and plastically deforming Stainless steel (SS) probe with different lengths**. The cross section is assumed to be 140 µm x280 µm for both cases. In FEA simulations, probes are modelled as cantilevers with rectangular cross section where one end is clamped, and the other end is free. A point load is applied at the free end. Yield strength (Stainless steel) and fracture strength (Silicon) were assumed to 1.25 GPa and 400 MPa, respectively.

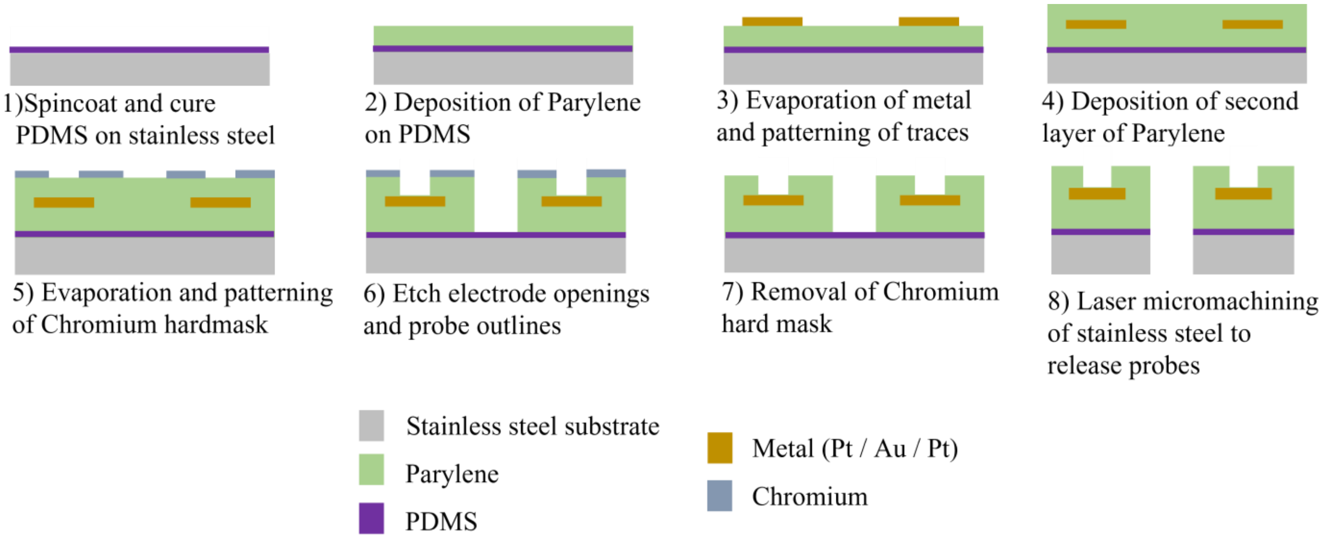

Figure S2 Novel fabrication process flow for monolithic integration of Parylene C encapsulated neural probe on stainless steel.

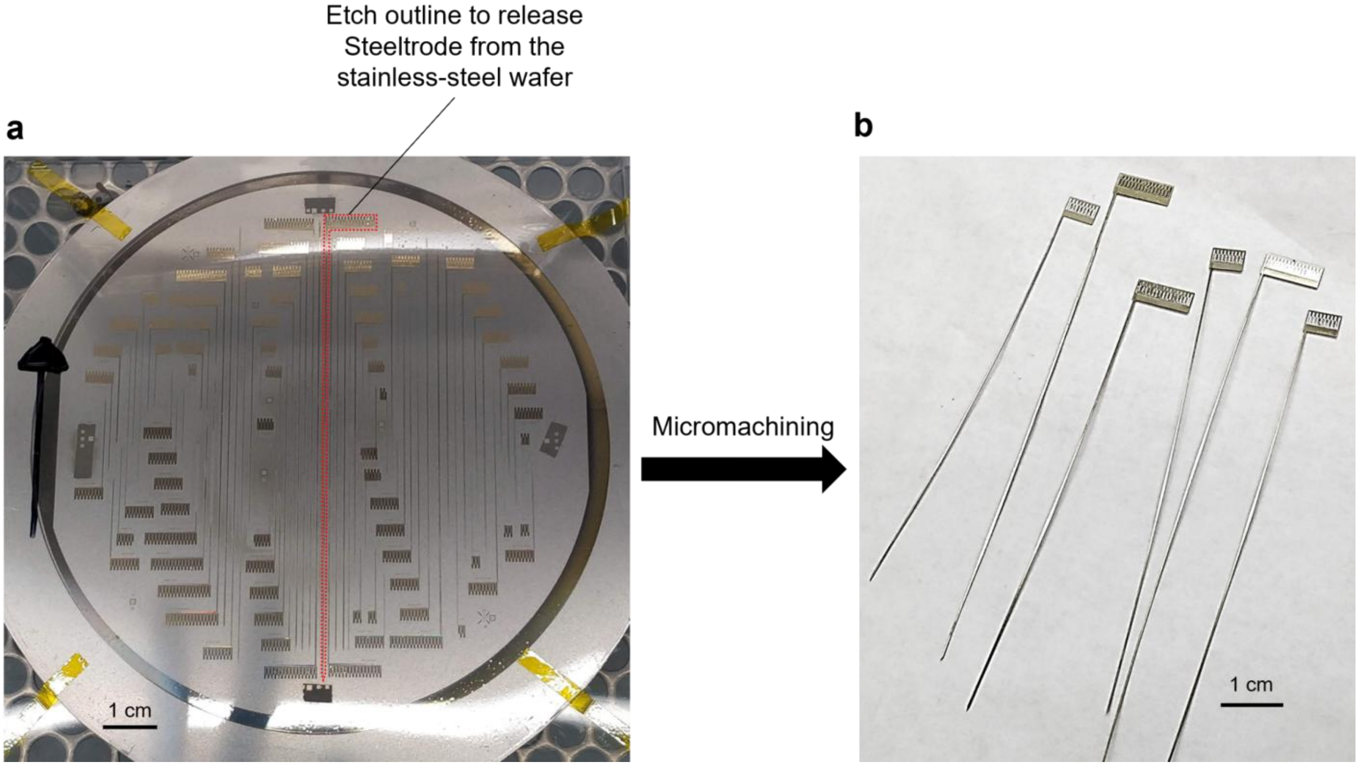

Figure S3. **Wafer release process of Microfabricated stainless steel neural probe. a** Photograph of a processed stainless steel wafer following deposition, patterning, and etching of insulation and metal layers using planar microfabrication techniques (see Methods for details). The red outline indicates the laser ablation path used to etch and release an individual steeltrode device from the wafer. **b** Steeltrodes after singulation via micromachining.

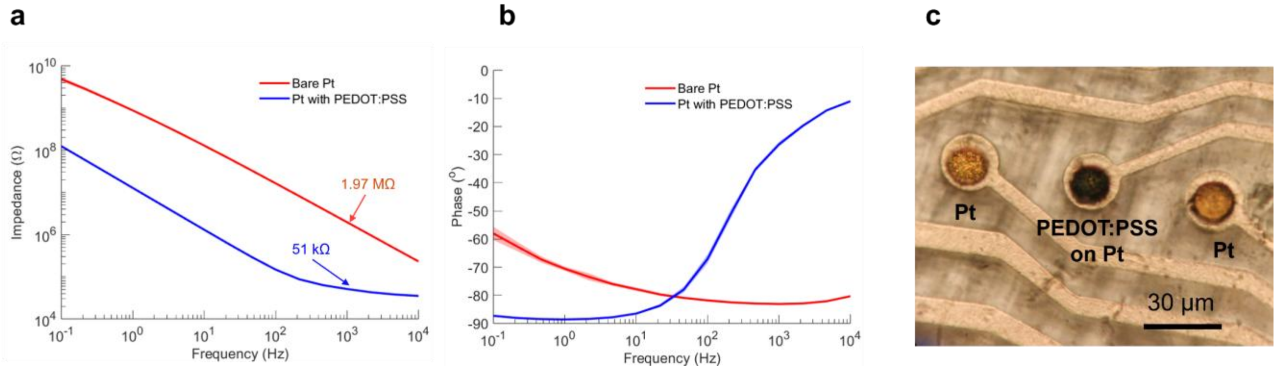

Figure S4 **Electrochemical impedance spectroscopy (EIS) measurements comparing bare Pt electrodes and PEDOT:PSS-coated electrodes**. Magnitude (mean ± SD) and phase (mean ± SD) of impedance spectra (N = 14 channels) shown in **a** and **b,** respectively. **c** Microscope image illustrating the coated and uncoated electrodes.
