# Supplementary material for: Robust minimally-invasive microfabricated stainless steel neural interfaces for high resolution recording
Source: Nat Commun. 2026 Jan 9;17:957. doi: 10.1038/s41467-025-67681-w (PMC12848124; doi:10.1038/s41467-025-67681-w)
Supplement: Supplementary file 2 — Reporting Summary [file 41467_2025_67681_MOESM2_ESM.pdf]

nature portfolio

Corresponding author(s): Maysam Chamanzar

Last updated by author(s): 05.22.2025

Reporting Summary

Nature Portfolio wishes to improve the reproducibility of the work that we publish. This form provides structure for consistency and transparency in reporting. For further information on Nature Portfolio policies, see our Editorial Policies and the Editorial Policy Checklist.

Statistics

For all statistical analyses, confirm that the following items are present in the figure legend, table legend, main text, or Methods section.

n/a	Confirmed	
☐	☒	The exact sample size (*n*) for each experimental group/condition, given as a discrete number and unit of measurement
☐	☒	A statement on whether measurements were taken from distinct samples or whether the same sample was measured repeatedly
☐	☒	The statistical test(s) used AND whether they are one- or two-sided<br>*Only common tests should be described solely by name; describe more complex techniques in the Methods section.*
☒	☐	A description of all covariates tested
☒	☐	A description of any assumptions or corrections, such as tests of normality and adjustment for multiple comparisons
☐	☒	A full description of the statistical parameters including central tendency (e.g. means) or other basic estimates (e.g. regression coefficient) AND variation (e.g. standard deviation) or associated estimates of uncertainty (e.g. confidence intervals)
☒	☐	For null hypothesis testing, the test statistic (e.g. F, t, r) with confidence intervals, effect sizes, degrees of freedom and P value noted<br>*Give P values as exact values whenever suitable.*
☒	☐	For Bayesian analysis, information on the choice of priors and Markov chain Monte Carlo settings
☒	☐	For hierarchical and complex designs, identification of the appropriate level for tests and full reporting of outcomes
☒	☐	Estimates of effect sizes (e.g. Cohen's *d*, Pearson's *r*), indicating how they were calculated

Our web collection on statistics for biologists contains articles on many of the points above.

Software and code

Policy information about availability of computer code

Data collection	All neural recording data presented in this work was collected with RHX Data Acquisition Software from Intan Technologies.
Data analysis	Local field potential (LFP) and multi-unit activity (MUA) data were analyzed using custom-developed MATLAB 2024a scripts. Non-human primate single-unit data were processed and analyzed using custom Python scripts built on the Mountainsort. Spike band data for rodents were analyzed and processed with Offline Sorter, v3.3.5, from Plexon Inc.

For manuscripts utilizing custom algorithms or software that are central to the research but not yet described in published literature, software must be made available to editors and reviewers. We strongly encourage code deposition in a community repository (e.g. GitHub). See the Nature Portfolio guidelines for submitting code & software for further information.

Data

Policy information about availability of data

All manuscripts must include a data availability statement. This statement should provide the following information, where applicable:

- Accession codes, unique identifiers, or web links for publicly available datasets
- A description of any restrictions on data availability
- For clinical datasets or third party data, please ensure that the statement adheres to our policy

The datasets generated and analyzed during the current study are available from the corresponding author upon reasonable request.

Research involving human participants, their data, or biological material

Policy information about studies with human participants or human data. See also policy information about sex, gender (identity/presentation), and sexual orientation and race, ethnicity and racism.

Reporting on sex and gender	N/A
Reporting on race, ethnicity, or other socially relevant groupings	N/A
Population characteristics	N/A
Recruitment	N/A
Ethics oversight	N/A

Note that full information on the approval of the study protocol must also be provided in the manuscript.

Field-specific reporting

Please select the one below that is the best fit for your research. If you are not sure, read the appropriate sections before making your selection.

☒ Life sciences ☐ Behavioural & social sciences ☐ Ecological, evolutionary & environmental sciences

For a reference copy of the document with all sections, see nature.com/documents/nr-reporting-summary-flat.pdf

Life sciences study design

All studies must disclose on these points even when the disclosure is negative.

Sample size	For the NHP experiments the sample size was n =1. Given the scarcity of NHPs and the time and cost it takes to maintain, train and prepare NHPs for experiments, using a single NHP is customary. However, we have repeated each neural recording experiments from the auditory cortex of the NHP at least m> 5 times.<br>For the rodent experiments, as no prior data were available to estimate effect sizes for this exploratory study, a formal a priori power analysis was not feasible. For each group/condition total 2-3 hemispheres were chosen from 4 different animals with the goal of evaluating the experimental approach and generating preliminary data for future, adequately powered studies.
Data exclusions	In rodent work, 2 additional animals were excluded from the final analysis due to physiological instability during the procedure, including irregular respiration and loss of reflex suppression despite adjustments in anesthesia.
Replication	All procedures were standardized and carried out using the same surgical setup, probe insertion approach and recording protocols across animals. Data acquisition and analysis were performed using pre-defined, automated scripts where possible to minimize subjective bias. For histological analyses, multiple sections (6–9 per hemisphere) were examined across three different condition to ensure consistency of observed patterns.
Randomization	Randomization was applied at key stages to minimize potential bias. During histological processing, brain sections were selected from anatomically matched regions without reference to experimental outcomes. From each hemisphere 6–9 sections were randomly selected per condition to ensure unbiased sampling.
Blinding	During data analysis, sample identifiers were anonymized so that experimenters were blinded to condition assignments where applicable. These steps were taken to ensure that sample handling, imaging, and analysis were not influenced by experimental expectations.

Reporting for specific materials, systems and methods

We require information from authors about some types of materials, experimental systems and methods used in many studies. Here, indicate whether each material, system or method listed is relevant to your study. If you are not sure if a list item applies to your research, read the appropriate section before selecting a response.

Materials & experimental systems

n/a	Involved in the study
☒	☐ Antibodies
☒	☐ Eukaryotic cell lines
☒	☐ Palaeontology and archaeology
☐	☒ Animals and other organisms
☒	☐ Clinical data
☒	☐ Dual use research of concern
☒	☐ Plants

Methods

n/a	Involved in the study
☒	☐ ChIP-seq
☒	☐ Flow cytometry
☒	☐ MRI-based neuroimaging

Animals and other research organisms

Policy information about studies involving animals; ARRIVE guidelines recommended for reporting animal research, and Sex and Gender in Research

Laboratory animals	For rodent studies, 3 month old Sprague Dawley rats of both sex were used. For non-human primate experiments, an adult macaque mulatta was used.
Wild animals	*Provide details on animals observed in or captured in the field; report species and age where possible. Describe how animals were caught and transported and what happened to captive animals after the study (if killed, explain why and describe method; if released, say where and when) OR state that the study did not involve wild animals.*
Reporting on sex	*Indicate if findings apply to only one sex; describe whether sex was considered in study design, methods used for assigning sex. Provide data disaggregated for sex where this information has been collected in the source data as appropriate; provide overall numbers in this Reporting Summary. Please state if this information has not been collected. Report sex-based analyses where performed, justify reasons for lack of sex-based analysis.*
Field-collected samples	*For laboratory work with field-collected samples, describe all relevant parameters such as housing, maintenance, temperature, photoperiod and end-of-experiment protocol OR state that the study did not involve samples collected from the field.*
Ethics oversight	All experiments on macaques were conducted in compliance with the Institutional Animal Care and Use Committee (IACUC) at the University of Pittsburgh. All rodent experiments conducted in this study received ethical approval from the Institutional Animal Care & Use Committee (IACUC) of Carnegie Mellon University.

Note that full information on the approval of the study protocol must also be provided in the manuscript.

Plants

Seed stocks	*Report on the source of all seed stocks or other plant material used. If applicable, state the seed stock centre and catalogue number. If plant specimens were collected from the field, describe the collection location, date and sampling procedures.*
Novel plant genotypes	*Describe the methods by which all novel plant genotypes were produced. This includes those generated by transgenic approaches, gene editing, chemical/radiation-based mutagenesis and hybridization. For transgenic lines, describe the transformation method, the number of independent lines analyzed and the generation upon which experiments were performed. For gene-edited lines, describe the editor used, the endogenous sequence targeted for editing, the targeting guide RNA sequence (if applicable) and how the editor was applied.*
Authentication	*Describe any authentication procedures for each seed stock used or novel genotype generated. Describe any experiments used to assess the effect of a mutation and, where applicable, how potential secondary effects (e.g. second site T-DNA insertions, mosiacism, off-target gene editing) were examined.*
